# Anticoagulation is not associated with an increased risk of variceal bleeding under systemic therapy for advanced HCC

**DOI:** 10.1016/j.jhepr.2024.101120

**Published:** 2024-05-16

**Authors:** Manon Allaire, Philippe Sultanik, Dominique Thabut

**Affiliations:** 1 AP-HP Sorbonne Université, Hôpital Universitaire Pitié-Salpêtrière, Service d’Hépato-gastroentérologie, Paris, France; 2 INSERM UMR 1138, Centre de recherche des Cordeliers, 75006 Paris, France; 3 Sorbonne Université, INSERM, Centre de recherche Saint-Antoine (CRSA), Institute of Cardiometabolism and Nutrition (ICAN), F-75012 Paris, France

To the Editor:

We have read with great interest the original research conducted by Ben Khaled *et al.*, which evaluated the risk of bleeding and thromboembolic events in patient with advanced hepatocellular carcinoma (HCC) treated with atezolizumab-bevacizumab (atezo/bev) and lenvatinib.^1^ The occurrence of acute variceal bleeding (AVB) was low, around 3% in the two cohorts of patients. Spleen size and the presence of large size varices were associated with AVB in univariate analysis, but no multivariate analysis was performed due to the low number of patients experiencing AVB. Interestingly, in this article, therapeutic anticoagulation (29% in the atezo/bev and 27% in the lenvatinib group) was not associated with an increased risk of AVB and gastrointestinal bleeding. This insight is particularly important, especially in the context of the perceived risk associated with anticoagulation, which might prevent some physicians from considering systemic therapies, such as atezo/bev, due to concerns about bleeding events. Our own series of 200 patients treated with atezo/bev in first line revealed a similar observation.^2^ To note, as for Imbrave150, all our patients benefited from upper endoscopy before starting atezo/bev treatment.^3^ Among the 20% of patients who underwent therapeutic anticoagulation, 46% presented with portal vein tumor thrombosis, 86% and 100% received adapted primary and secondary AVB prophylaxis, respectively, and yet, anticoagulation was not associated with increased mortality (adjusted hazard ratio [aHR] 0.75, 95% CI 0.47-1.20) or higher risk of AVB (aHR 1.34, 95% CI 0.48-3.72) in univariate Cox analysis. However, in our series, AVB incidence was higher (12% at 12 months) and was independently associated with the presence of portal vein tumor thrombosis (aHR 3.25, 95% CI 1.16-9.07), history of AVB <6 months (aHR 4.32, 95% CI 1.17-15.92) and varices of any size (aHR 3.22, 95% CI 1.02-10.14) in Cox multivariate analysis. In the series of Ben Khaled *et al.*, the percentage of AVB prophylactic therapy was 34% in the patients receiving atezo/bev, which seems to be low at first sight, but the proportion of patients receiving adapted primary/secondary prophylaxis for AVB is missing in this series (meaning non-selective beta-blockers and/or ligation in the presence of varices).^1^ However, we believe that if AVB prophylaxis is meticulously managed, anticoagulation should not preclude the use of systemic therapy, especially atezo/bev. Achieving this, of course, necessitates thorough screening for portal hypertension with systematic upper endoscopy as Baveno criteria do not appear to be performant in patients with advanced HCC.^4^ The recent AASLD guidelines propose that patients with large varices should likely undergo at least one session of band ligation prior to atezo/bev initiation, although carvedilol may also be effective.^5^ Rather than systematic band ligation in patients with large varices, we advocate for the consideration of non-selective beta-blockers as primary prophylaxis in all patients with esophageal varices, regardless of their size.^6^^,^^7^ First, because it can be difficult to distinguish between small- and medium-sized esophageal varices and, second, because portal hypertension levels might be impacted by HCC treatment, and third because band ligation might delay the initiation of the treatment due to the fear of bleeding under bevacizumab after the procedure.

Hence, these findings defy the notion that atezo/bev for advanced HCC should be contraindicated in patients undergoing therapeutic anticoagulation, provided that portal hypertension screening is meticulously conducted, and AVB prophylaxis is appropriately administered.

## Financial support

The authors did not receive any financial support to produce this manuscript.

## Conflict of interest

The authors of this study declare that they do not have any conflict of interest.

Please refer to the accompanying ICMJE disclosure forms for further details.

## Authors’ contributions

Manon Allaire wrote the letter. Philippe Sultanik and Dominique Thabut revised and approved the final version of the letter. All authors approved the final version of the manuscript. Dominique Thabut is acting as the submission's guarantor.
